# Determination for a suitable ratio of dried black pepper and cinnamon powder in the development of mixed-spice ice cream

**DOI:** 10.1038/s41598-022-19451-7

**Published:** 2022-09-06

**Authors:** Pajaree Aumpa, Amita Khawsud, Taruedee Jannu, Gerry Renaldi, Niramon Utama-Ang, Shitapan Bai-Ngew, Ponjan Walter, Rajnibhas Sukeaw Samakradhamrongthai

**Affiliations:** 1 Food Science and Technology Program, Faculty of Agro-Industry, Prince of Songkla University, Hat Yai, 90110 Songkhla Thailand; 2 Agro-Industrial Product Development Research Unit, Faculty of Agro-Industry, Prince of Songkla University, Hat Yai, 90110 Songkhla Thailand; 3 Division of Product Development Technology, Faculty of Agro-Industry, Chiang Mai University, Chiang Mai, 50100 Thailand; 4 Cluster of Innovative Food and Agro-Industry, Chiang Mai University, Chiang Mai, 50100 Thailand

**Keywords:** Analytical chemistry, Mathematics and computing

## Abstract

Black pepper powder (BPP) and cinnamon powder (CP) are traditionally used as food ingredients and can apply related to developing a functional product. In this study, BPP and CP were used as an ingredient in mixed-spice ice cream (MSIC). The physicochemical properties, textural properties, and sensory qualities were investigated as key points. BPP (0.51–17.49 g) and CP (8.79–51.21 g) were combined using a central composite design (CCD) with 2 centerpoints. The optimized BPP and CP for the MSIC were 15.00 g and 34.00 g, which exhibited firmness and overrun at 3210.65 ± 105.74 g.force and 61.63 ± 0.60%. The MSIC with optimized BPP and CP also provides high bioactive compounds and antioxidant activities with cinnamaldehyde and piperine as flavor characteristics. The findings indicated that BPP and CP can enhance the functional properties and provide alternative flavors in the food product, providing an innovative approach to deliver health-beneficial combinations for consumer satisfaction.

## Introduction

Many dairy products can act as successful carriers in delivering phytochemicals and nutrients that exhibit health-beneficial effects in our system of nutrition and food^1^. Ice cream is one of the most consumed dairy products in the world. However, ice cream is commonly poor in natural compounds exhibiting polyphenol, antioxidants, and even color. An interesting field of research to investigate is the possibility to enhance and increase the nutritional values of ice cream using ingredients beneficial for health, such as herbs and spices^2,3^.

Herbs and Spices are natural additives that contribute to creating flavor in foods. In addition to contributing to the taste of food, spices exhibit nutritional value and health-related beneficial properties. The incorporation of herbs, spices, and/or extracts to various dairy products, including ice cream, can make these products suitable carriers for nutraceuticals^4,5^.

Black pepper (*Piper nigrum* L.) and cinnamon (*Cinnamomum zeylanicum* Linn.) are well-known spices that are used for various medicinal and flavor-enhancing purposes throughout the world. Black pepper is a medicinal plant from the family Piperaceae which is currently employed in many aspects of the food and pharmaceutical industries. Cinnamon is a prominent spice used in the aroma and flavor industry that can be applied to food, fragrance, and medicinal products. They contained various bioactive compounds such as carotenoids (lutein and beta-carotene) and flavonoids (catechin, myricetin, and quercetin), which plays a role in the antioxidant, antiallergic, and anti-inflammatory activities^6^. The monoterpenes including linalool, α-phellandrene, limonene, α- and β-pinene, myrcene, methylpropanal, and 3-methylbutanal are the important odorants of black pepper, while mouldy and musty off-flavor is 2,3-diethyl-5-methylpyrazine and 2-isoprophyl-3-methoxypyrazine^7^. While cinnamon volatile flavor was cinnamaldehyde, copaene, caryophyllene, and bornyl acetate^8^.

The study on the incorporation of black pepper powder (BPP) and cinnamon powder (CP) into an ice cream product can be a novelty and innovative way to deliver health-beneficial compounds to consumers. Thus, the objectives of this study were to optimize the amount of BPP and CP for mixed spice ice cream (MSIC) and evaluate its effect on the physicochemical properties and sensory qualities.

## Materials and methods

### Materials

All the ice cream ingredients were purchased from Tesco Lotus, Thailand: pasteurized milk (CP Meiji Co., Ltd., Thailand); skim milk powder (Dairy farm, New Zealand); whipped cream (original Reddi wip, USA); sugar (Mitr Phol, Thailand); BPP and CP (McCormick, USA). Guar gum was obtained from Chemipan Corporation Co., Ltd., Bangkok, Thailand. All ice cream samples were prepared in the pilot plant at the Faculty of Agro-Industry, Prince of Songkla University (Hat Yai Campus), Songkhla, Thailand.

### Chemicals

The chemicals used in this experiment were: methanol (PubChem CID 887), acquired from Avantor Performance Materials, LLC., Poland; aluminium chloride (PubChem CID 24564), FeCl_3_.6H_2_O (PubChem CID 16211236), sodium carbonate (PubChem CID 10340), and sodium nitrite (PubChem CID 233668193), purchased from Kemaus, Australia; Folin–Ciocalteau reagent and NaOH (PubChem CID 14798) procured from Loba Chemie Pvt. Ltd., India; ABTS (PubChem CID 16240279), DPPH (PubChem CID 57654141), potassium persulfate (PubChem CID 24412), and TPTZ (PubChem CID 77258) acquired from Sigma-Aldrich, Chemie GmbH, Germany. All chemicals were analytical grade.

### MSIC preparation

The MSIC preparation followed the preparation method of reduced-fat ice cream from Samakradhamrongthai et al.^9,10^ with slight modification. The constant ingredients of MSIC consisted of pasteurized milk (62.50%), whipped cream (22.20%), sugar (8.58%), inulin (4.02%), skim milk powder (2.30%), and guar gum (0.30%). The BPP and CP were varied in the range of 3.00–15.00 g and 15.00–45.00 g using the central composite design with 2 center points (Supplementary Table A). The BPP and CP were mixed homogeneously and 0.1% of mixed spice powder (100 µm) was then blended with other ingredients to produce MSIC.

Whipped cream and pasteurized milk were heated in a pan at 50 °C with stirring. The dry ingredients (sugar, guar gum, skim milk powder, and mixed spice powder) were mixed thoroughly, added into the liquid ice cream mix when the temperature reached 60 °C and stirred into a homogeneous mixture using a laboratory homogenizer (PRO250 Homogenizer, Thomas 1204B63, Thomas Scientific, USA) for 2 min at 3350 g. The liquid ice cream mixes were pasteurized for 30 s at 80 ± 1 °C, homogenized using a laboratory homogenizer (PRO250 Homogenizer, Thomas 1204B63, Thomas Scientific, USA) at 3350 g for 5 min at 60 °C and then cooled to 20 °C. To ensure the complete hydration of the ingredients, the ice cream mixes were stored for 24 h at 4 °C. The ice cream mixes were frozen for 30 min in a batch ice cream laboratory freezer (Marchcool, Thailand). The ice cream samples were separated into 2 portions, in a 175 mL plastic container for physical properties and ice cream characteristic analysis, and 10 mL plastic cups for sensory evaluation. All samples were taken to harden at − 30 °C for 24 h and stored at − 18 °C before the analysis.

### Color measurement

After the storage at − 18 °C, the MSIC samples were immediately placed in a chamber in which they were uniformly illuminated from all directions. Color measurement was performed using a Color Quest XE Colorimeter (Color Global, USA), according to CIEL*a*b* system, using spectral reflectance for calibration, illuminant D65, and an observation angle of 108. The L* (lightness), a* (red intensity), and b* (yellow intensity) values were measured at six different points for each sample. The color was measured in triplicate on each sample. The whiteness index (WI) was calculated with the following equation:

$$ {\text{WI}} = 100 - \sqrt {( {100 - L^{*} } )^{2} + (a^{*} )^{2} + (b^{*} )^{2} } $$

### Texture analysis

The texture profile was assessed according to Kaleda et al.^11^ with modification. The analysis was performed at 25 °C using a TA.XT plus Texture Analyzer (Stable Micro System, UK), equipped with a vertical stainless-steel sheet (30 × 1 mm). The firmness of ice cream samples (500 mL) was measured by penetration of a vertical sheet into the ice cream to a depth of 20 mm at a constant speed of 20 mm/min. For every batch, 10 different samples were analyzed at three different places in the ice cream box.

### Apparent viscosity

The concentric cylinder viscometer (model LVT, Brookfield, Stoughton, MA) was used to measure the apparent viscosity^9^. The apparent viscosity of the ice cream mix (400 mL) at 5 ± 0.5 °C after 48 h of aging was measured in triplicate with a spindle number 63 at a shear rate of 102/s. One measurement was taken per mix. The ice cream mix temperature was controlled at 5 ± 0.5 °C using a jacketed beaker connected to a refrigerated bath (Lauda RCS-20D; Lauda-Konigshofen, Germany) circulating a 50% aqueous solution of ethylene glycol.

### Melting rate

The melting rate of ice cream samples was measured using 175 mL of ice cream sample stored at − 18 °C for 7 days^12^. The samples were then placed on a 1 mm stainless steel mesh at ambient temperature (20 ± 1 °C). The weight of the melted ice cream was recorded every 10 min until the ice cream was melted completely. A plot of the percentage of melted ice cream versus time was created with the slope of the linear part of the plot indicating the melting rate (g/min). Measurement was done in triplicate.

### Overrun

The overrun of the MSIC was measured and performed by weighing the ice cream mix in a fixed volume container (175 mL) separately^9^. The overrun measurement was carried out in triplicate and measured by the following equation:

$$ \% {\text{ Overrun }} = \frac{Weight\, of\, ice\, cream\, mix - Weight\, of\, ice\, cream}{{Weight\, of\, ice\, cream}} \times 100 $$

### Total phenolic content (TPC) and antioxidant activities

#### Sample extraction

To analyze TPC and antioxidant activities of MSIC, the ice cream sample was mixed with methanol at a ratio of 1:20, then mixed thoroughly using a magnetic stirrer for 15 min. The MSIC extract was then filtered using Whatman filter paper No. 1 and prepared into a methanolic extract (1 mg/mL). The prepared filtrate was stored in an amber bottle at 4 °C until the further determination of TPC and antioxidant activities. All analysis was calculated from the Trolox calibration curve and expressed as mg Trolox equivalent/g of sample^9,13^.

#### Total phenolic content (TPC)

The TPC of the MSIC was determined using the Folin–Ciocalteau method. The 100 μL of methanolic extract was mixed with 0.5 mL of Folin–Ciocalteau phenol reagent and 2 mL of Na_2_CO_3_ (20% w/v). The mixed solution was allowed to stand for a further 30 min in the dark. The absorbance of the solution was measured at 750 nm.

#### Determination of antioxidant activities

DPPH radical scavenging activity was measured by mixing 200 μL methanolic extract with 3.8 mL of 0.5 mM DPPH methanolic solution (0.5 mM), and allowing it to stand in the dark for 30 min. The absorbance was immediately measured at 517 nm.

Ferric reducing antioxidant power (FRAP) was determined using 0.25 mL of methanolic extract mixed with 0.25 mL of FRAP reagent and incubated at 37 °C for 20 min. Subsequently, the mixture was centrifuged at 3350 g for 10 min. The supernatant (1 mL) was mixed with 1 mL of distilled water and 0.2 mL of 1% ferric chloride and the absorbance was measured at 593 nm.

An ABTS assay was conducted using a spectrophotometer (Biochrom Libra S22, Biochrom, UK). A working solution was obtained by mixing ABTS stock solution (7 mM ABTS in water and 2.45 mM potassium persulfate, 1:1). The solution was kept for 12–16 h at room temperature in the dark. The MSIC extract (150 μL) was mixed with the ABTS· + solution (2.85 mL) and measured at 734 nm.

### Gas chromatography analysis of the main aroma of BPP and CP from MSIC

Before analysis, the volatiles from MSIC were extracted by carboxen/polydimethylsiloxane fiber (CAR/PDMS). A 2 g of samples were extracted with ethanol in 20 mL vials. The extraction was performed at 60 °C for 60 min. The bounded volatile compounds were analyzed by gas chromatography (GC). The GC analysis was performed using 7890B and mass Spectrometry (MS) was performed by using an Agilent 5977B system (Agilent Technologies, Inc., USA). The chromatographic column was HP-5msUI (30 m × 0.25 mm ID and 0.5 μm film thickness, Model 19091S-433I, Agilent Technologies, Inc., USA) and helium carrier gas (at a flow rate of 29.0 mL/min) was used for both analyses. A 1 μL of sample headspace was injected in split mode with a split ratio at 25:1. The injection port temperature was set at 280 °C. The temperature program was started at 50 °C for 1 min and then increased to 70 °C at 5 °C/min for 2 min. After that, the temperature was increased to 100 °C at 3 °C/min for 1 min, then the temperature was increased to 130 °C at 15 °C/min 1 min. The temperature was increased further to 140 °C at 4 °C/min and maintained for 1 min, then the temperature was increased again to 280 °C at 9 °C/min for 2 min. Finally, the temperature was increased to 300 °C at 10 °C/min and maintained for 5 min^14,15^. The relative content of the identified volatile compounds was calculated based on 1 μL of hexanal solution as an external standard reference to create a calibration curve. The relative amount was calculated from the constant ratio of hexanal and effective carbon number (ECN) of the identified volatile compounds.

### Sensory evaluation

The sensory evaluations of developed ice cream (8 formulations) and final formulation were evaluated by sixty and four hundred participants according to the Declaration of Helsinki guidelines. All sensory evaluations were performed under the approval of the Office of Human Research Ethics Committee, Health Sciences, Prince of Songkla University (Approval No: HSc-HREC-62-005-1-1). The consumer was recruited from students and staff from Prince of Songkla University, Hat Yai, Thailand, and written informed consent was obtained from all participants before the evaluation. All participants signed and returned the consent form to the research team, and the consent of the participants was ensured before participating in the study. The MSIC was presented in disposable closed-lid plastic cups, each coded with a three-digit number which was evaluated by each consumer in a monadic order, following a balanced design^16^. The MSIC sample for sensory evaluation was retained under 4 ± 1 °C until the assessment was initiated. The MSIC was evaluated using a 9-point hedonic scale on the appearance, black pepper aroma, cinnamon aroma, melt in mouth, black pepper flavor, cinnamon flavor, sweetness, creaminess, oiliness, viscosity, spiciness, and overall liking.

### Statistical analysis

All measurements were done in triplicate and reported as a mean ± standard deviation. The statistical analysis was conducted using SPSS 17.0 (SPSS Inc., IBM Corp., IL, USA) using Duncan’s multiple range test (DMRT) at the significance level < 0.05. The response surface methodology (RSM) was applied to optimized BPP and CP using a regression equation by Design Expert 13 (Stat Ease Inc., MN, USA), with the significant responses selected to generate the optimized amount of BPP and CP for MSIC.

## Results and discussion

### Color measurement of MSIC

The incorporation of BPP and CP in the MSIC significantly affected the physical properties of the MSIC (Table 1). The L*, a*, and b* were in the range of 83.77–87.91, 1.19–2.09, and 15.84–18.40, respectively. Increasing the amount of BPP and CP significantly decreased the L* value of the MSIC, while increasing the a* and b* values. The changes of color intensity happened because the spices contained several pigment compounds such as carotenoids and flavonoids^17^, which are yellow, red, and brown and were responsible for the alteration of color properties in the products. The carotenoids and flavonoids of black pepper and cinnamon are lutein and beta-carotene, and catechin, myricetin, and quercetin, which showed antioxidant, antiallergic, and anti-inflammatory activities^6^. However, the addition of spices was not significantly affected to whiteness index (WI) of developed ice creams (*p* > 0.05). These findings suggest that the addition of spices into a product with an unpigmented color base can alter the color into a darker tone with tints of yellow, red, and brown. These results were in the same direction as many pieces of research that added spices and extract them into ice cream^5,18–20^.

**Table 1:** Physical properties, textural properties, total phenolic content, and antioxidant activities of MSIC.

TRT	Color value				Firmness (g.force)	Apparent viscosity (cP)	Melting rate (g/min)	Overrun (%)	TPC (mg TE/g sample)	Antioxidant activities (mg TE/g sample)		
										DPPH	FRAP	ABTS
	L*	a*	b*	WI^ns^								
1	87.91 ± 0.13^a^	2.06 ± 0.05^a^	18.40 ± 0.12^a^	77.89 ± 0.05	2325.67 ± 387.2^c^	1355.00 ± 1.00^b^	0.52 ± 0.01^a^	65.97 ± 1.07^b^	325.66 ± 2.92^e^	155.47 ± 0.92^c^	44.93 ± 4.37^d^	1.02 ± 0.04^a^
2	86.60 ± 0.05^d^	1.81 ± 0.06^b^	17.51 ± 0.03^b^	77.88 ± 0.06	676.09 ± 12.21^fg^	1234.67 ± 4.16^d^	0.27 ± 0.01^f^	46.80 ± 2.10^e^	290.25 ± 1.06^f^	126.79 ± 1.13^e^	135.49 ± 1.54^a^	0.95 ± 0.03^a^
3	87.02 ± 0.05^c^	1.66 ± 0.02^c^	17.66 ± 0.11^b^	78.02 ± 0.11	1460.53 ± 26.58^d^	1329.67 ± 0.58^c^	0.43 ± 0.01^c^	32.37 ± 3.02^g^	216.72 ± 2.53^i^	130.23 ± 0.34^d^	32.07 ± 1.13^f^	0.57 ± 0.03^d^
4	87.03 ± 0.18^c^	1.56 ± 0.07^d^	16.76 ± 0.12^e^	78.75 ± 0.09	1150.91 ± 26.93^e^	506.30 ± 0.96^f^	0.32 ± 0.01^e^	82.83 ± 0.95^a^	227.49 ± 1.77^h^	115.38 ± 1.05^f^	85.79 ± 1.03^b^	0.40 ± 0.02^g^
5	86.87 ± 0.10^cd^	1.67 ± 0.02^c^	16.99 ± 0.06^d^	78.46 ± 0.06	2679.10 ± 76.23^b^	912.67 ± 1.53^e^	0.27 ± 0.01^f^	37.93 ± 0.91^f^	484.40 ± 3.62^a^	113.05 ± 0.67^f^	75.96 ± 3.71^c^	0.48 ± 0.01^e^
6	86.15 ± 0.08^e^	1.55 ± 0.01^d^	16.64 ± 0.10^e^	78.29 ± 0.03	2610.58 ± 154.13^b^	442.60 ± 9.42^h^	0.46 ± 0.01^b^	55.30 ± 0.40^c^	244.59 ± 5.26^g^	154.05 ± 2.71^c^	25.29 ± 1.45^g^	0.44 ± 0.01^f^
7	85.79 ± 0.01^f^	1.19 ± 0.05^e^	16.28 ± 0.11^f^	78.36 ± 0.04	548.53 ± 5.38^g^	479.73 ± 1.62^g^	0.35 ± 0.02^d^	38.37 ± 1.12^f^	374.06 ± 3.81^b^	158.31 ± 2.00^b^	28.83 ± 1.07^fg^	0.61 ± 0.01^c^
8	83.77 ± 0.55^g^	2.09 ± 0.13^a^	15.84 ± 0.17^g^	77.23 ± 0.07	874.41 ± 7.69^f^	480.37 ± 0.40^g^	0.31 ± 0.01^e^	51.83 ± 0.93^d^	348.69 ± 4.57^d^	168.15 ± 1.82^a^	36.94 ± 1.55^e^	0.48 ± 0.01^e^
9	87.78 ± 0.16^a^	1.79 ± 0.04^b^	18.17 ± 0.29^c^	78.03 ± 0.06	4519.10 ± 2.18^a^	1722.67 ± 2.18^a^	0.23 ± 0.01^g^	47.20 ± 0.35^e^	356.43 ± 0.58^c^	166.81 ± 0.58^a^	42.20 ± 0.06^d^	0.74 ± 0.02^b^
10	86.42 ± 0.08^b^	1.70 ± 0.08^b^	17.08 ± 0.05^c^	78.11 ± 0.08	4530.67 ± 8.96^a^	1723.77 ± 8.96^a^	0.23 ± 0.01^g^	47.77 ± 0.06^e^	357.04 ± 0.84^c^	165.58 ± 1.77^a^	42.44 ± 0.40^d^	0.74 ± 0.01^b^
p-value	< 0.001	< 0.001	< 0.001	> 0.05	< 0.001	< 0.001	< 0.001	< 0.001	< 0.001	< 0.001	< 0.001	< 0.001

The different superscript letter in the same column indicated the significant difference (p < 0.05).

### Firmness and apparent viscosity of MSIC

The firmness and apparent viscosity of the MSIC were in the range of 548.53–4519.10 g. force and 442.60–1723.77 cP (Table 1). All the MSIC containing different amounts of BPP and CP exhibited significant differences in firmness and apparent viscosity. This indicated that the addition of spices can affect the firmness and apparent viscosity of MSIC. The results also corresponded with the investigations of Nawal Galal et al.^21^ and Zuha et al.^22^ on functional frozen dairy product enrichment with fruits and vegetables. The addition of particles (fruits, vegetables, and spices) into frozen dairy products decreased the firmness and increased the apparent viscosity. In this research, the alteration of firmness and apparent viscosity happened because BPP and CP released volatile oil during the process into the ice cream mixture which contributed to the differentiation of the firmness and apparent viscosity^9,19^. Changes in firmness and apparent viscosity were also found when fermented pepper powder and silver ear mushroom powder were added to ice cream because the volatile compounds and solid content from additional particles or powders can influence the initial and gradual growth of ice crystals and interrupt product native thermodynamic instability^17,23^.

### Melting rate and overrun

The melting rate and overrun of MSIC were significant in the range of 0.23–0.52 g/min and 32.37–82.83% (Table 1). Increasing BPP and CP mixed powders increased the melting rate. This finding is insinuated and revealed that the addition of fiber powder into an ice cream matrix can increase thermal diffusion when the product is exposed to the environment and absorbs heat^24,25^. Moreover, the overrun in this research was found to be in the same direction as the melting rate. The increased overrun was also related to increasing mixed spice in the MSIC as shown in the results from Table 1 because the mixed spice released volatile oil compounds. Ice cream mixture which contains more volatile oil can exhibit higher apparent viscosity which affects the fluid laminar stretch of the mixture and assists to produce air cells with a more durable surface^26^. The overrun of MSIC was higher than the evaluation of herbal and menthol ice cream from Patil et al.^27^ when the mixed spice was increased. Moreover, the overrun declination was affected by the increase in BPP and CP amount, which corresponds to the findings from Vedashree et al.^20^ on the result of overrun from herbal ice cream. The decreasing overrun percentage of also affected by the interference from powder or particles ingredients in the ice cream matrix. It was also found that the incorporation of BPP and CP into the ice cream matrix can disrupt air cell lamellae and delay the incorporation of air into the ice cream matrix resulting in the decreasing overrun in ice cream^27,28^.

### Total phenolic content (TPC) and antioxidant activities

The variation of BPP and CP showed a significant effect on TPC and antioxidant activities of MSIC (Table 1). The TPC of the MSIC was in the range of 216.72–484.40 mg TE/g sample. The TPC in MSIC was detected in high amounts because BPP and CP contain both water-soluble and lipid-soluble polyphenol compounds such as epicatechin, catechin, trans-ferulic acid, and procyanidin B2^29^. Polyphenol compounds can also affect antioxidant activities which are unique parameters that measure the strength of a sample to quench free radicals and their preservative properties on lipid components from deterioration. Moreover, the addition of spices into food products such as BPP and CP can enhance antioxidant activities which suggest a positive correlation between the phenolic content and the antioxidant activity of spices^18^.

In this study, the antioxidant activity of developed ice cream was measured by three different methods namely DPPH, FRAP, and ABTS. The DPPH and ABTS assays are routinely used because it was easy to perform^30^. While FRAP was used to measure the ability of electron transfer from antioxidant to radical^30^. The variation of BPP and CP showed significant antioxidant activities using DPPH, FRAP, and ABTS assay in the range of 113.05–168.15, 25.29–135.49, and 0.40–1.02 mg TE/g sample, respectively. The increase in antioxidant activities was observed to be 3–5 times in the DPPH assay, 1–5 times in FRAP assay, and 2–5 times in ABTS assay with the addition of BPP and CP at 0.1% into ice cream. High concentrations of polyphenols and antioxidant activities from BPP and CP were the major contributors to high antioxidant activities in MSIC. The improvement in the antioxidant activity of the experimental sample was attributed to the incorporation of polyphenols, piperine, and cinnamaldehyde which have been proved to have high antioxidant properties and delivered a positive linear relationship correlation between antioxidant activity and total phenolic content^31,32^.

### Main aroma compound of BPP and CP from MSIC

The eight main volatile compounds in MSIC were benzaldehyde (12.69–25.39 µg/g sample), cinnamaldehyde (386.94–773.05 µg/g sample), β-pinene (4.62–27.34 µg/g sample), limonene (1.98–59.2 µg/g sample), bornyl acetate (1.44–2.81 µg/g sample), cinnamyl acetate (2.11–4.33 µg/g sample), β-caryophyllene (2.31–46.69 µg/g sample), and piperine (12.69–25.39 µg/g sample). Increasing the BPP can increase β-pinene, limonene, β-caryophyllene, and piperine whereas the increasing CP can increase benzaldehyde, cinnamaldehyde, bornyl acetate, and cinnamyl acetate due to these two different groups comprising the aromatic characteristics of black pepper and cinnamon. Those results were also indicated that the incorporation of BPP and CP in different amounts into the MSIC formulation also affected the content of these compounds significantly^4,33^ as is shown in Table 2. In addition, there was a fluctuation in β-caryophyllene analysis that can be observed in MSIC. This occurred because β-caryophyllene can be identified in both black pepper and cinnamon. It is commonly identified as the main active ingredient of black pepper while only an insignificant compound in cinnamon. Therefore, the intensity of β-caryophyllene can be noticeably detected due to its high threshold perception that can enhance the overall β-caryophyllene in MSIC.

**Table 2:** Main volatile compounds content from BPP and CP in MSIC.

TRT	Main volatile compound content (µg/g sample)							
	Benzaldehyde	β-Pinene	Limonene	Cinnamaldehyde	Bornyl acetate	Cinnamyl acetate	β-Caryophyllene	Piperine
1	21.49 ± 0.05^c^	11.53 ± 0.28^f^	19.49 ± 0.10^f^	655.25 ± 0.64^d^	2.37 ± 0.04^c^	3.61 ± 0.45^d^	15.62 ± 0.08^f^	21.49 ± 0.05^f^
2	12.94 ± 0.06^g^	26.73 ± 0.61^b^	58.45 ± 0.04^b^	392.70 ± 0.60^h^	1.45 ± 0.04^f^	2.15 ± 0.55^h^	45.70 ± 0.14^b^	12.94 ± 0.06^b^
3	24.34 ± 0.23^b^	6.73 ± 0.03^h^	7.27 ± 0.06^h^	736.52 ± 0.58^b^	2.72 ± 0.05^b^	4.05 ± 0.14^b^	6.37 ± 0.04^h^	24.34 ± 0.23^h^
4	19.43 ± 0.10^e^	15.35 ± 0.21^d^	29.43 ± 0.38^d^	589.48 ± 0.01^f^	2.17 ± 0.03^d^	3.27 ± 0.05^f^	23.45 ± 0.05^d^	19.43 ± 0.10^d^
5	25.39 ± 0.01^a^	4.62 ± 0.02^i^	1.98 ± 0.02^i^	773.05 ± 0.58^a^	2.81 ± 0.01^a^	4.33 ± 0.01^a^	2.31 ± 0.01^i^	25.39 ± 0.01^i^
6	16.38 ± 0.11^f^	20.91 ± 0.09^c^	42.96 ± 0.12^c^	496.70 ± 0.15^g^	1.80 ± 0.01^e^	2.76 ± 0.44^g^	34.39 ± 0.08^c^	16.38 ± 0.11^c^
7	12.69 ± 0.12^g^	27.34 ± 0.03^a^	59.23 ± 0.10^a^	386.94 ± 1.53^i^	1.44 ± 0.03^f^	2.11 ± 0.04^h^	46.69 ± 0.03^a^	12.69 ± 0.12^a^
8	21.50 ± 0.45^c^	10.79 ± 0.10^g^	17.53 ± 0.06^g^	667.49 ± 1.00^c^	2.37 ± 0.11^c^	3.73 ± 0.06^c^	14.28 ± 0.03^g^	21.50 ± 0.45^g^
9	19.89 ± 0.01^d^	14.58 ± 0.02^e^	26.96 ± 0.01^e^	604.55 ± 0.08^e^	2.19 ± 0.03^d^	3.36 ± 0.01^e^	21.69 ± 0.01^e^	19.89 ± 0.01^e^
10	19.93 ± 0.06^d^	14.57 ± 0.03^e^	26.95 ± 0.03^e^	604.44 ± 0.04^e^	2.20 ± 0.01^d^	3.31 ± 0.02^ef^	21.71 ± 0.02^e^	19.93 ± 0.06^e^
p-value	< 0.001	< 0.001	< 0.001	< 0.001	< 0.001	< 0.001	< 0.001	< 0.001

The different superscript letter in the same column indicated the significant difference (p < 0.05).

### Sensory evaluation on MSIC

The sensory rating score of MSIC revealed that BPP and CP affected color, cinnamon aroma, and pepper aroma insignificantly whereas the rest of the sensory rating score of MSIC was significantly different (Table 3). The insignificant sensory rating score of color occurred because the panels only considered product color with the naked eye which provided limited resolution compared to the colorimeter. It is not unusual for statistical differences to show insignificance. Furthermore, the insignificance of cinnamon and pepper aroma from ice cream was possible because the serving temperature was lower than room temperature, resulting in the aroma release being below the panel's perceptual threshold and causing the panel to be unable to detect the difference in aroma intensity^33,34^.

**Table 3:** Hedonic level of MSIC.

TRT	Appearance	Color^NS^	Cinnamon aroma^NS^	Pepper aroma^NS^	Melting in mouth	Cinnamon flavor	Pepper flavor	Sweetness	Creaminess	Oiliness	Viscosity	Spiciness	Overall liking
1	6.9 ± 0.7^abcde^	7.0 ± 0.9	6.2 ± 1.1	5.9 ± 1.3	5.0 ± 0.7^e^	5.6 ± 0.8^c^	6.1 ± 1.1^b^	5.4 ± 0.8^c^	6.5 ± 0.4^cd^	6.5 ± 1.1^c^	6.5 ± 1.0^c^	7.2 ± 0.8^a^	6.0 ± 1.2^c^
2	7.1 ± 0.6^abc^	7.0 ± 0.4	6.0 ± 0.3	5.7 ± 1.3	6.0 ± 0.5^c^	7.2 ± 0.9^a^	6.3 ± 0.9^ab^	7.0 ± 0.9^a^	6.5 ± 0.9^d^	6.5 ± 1.0^c^	6.5 ± 0.8^c^	7.1 ± 1.1^a^	7.1 ± 1.4^a^
3	6.7 ± 0.6^cde^	7.3 ± 0.4	6.3 ± 0.5	5.9 ± 1.0	5.6 ± 0.9^d^	6.5 ± 1.1^b^	4.6 ± 0.7^e^	6.8 ± 0.9^ab^	7.0 ± 0.2^abc^	7.0 ± 0.8^ab^	7.0 ± 0.8^ab^	5.1 ± 0.9^d^	5.1 ± 0.5^d^
4	6.7 ± 1.0^de^	7.1 ± 0.4	6.0 ± 1.1	5.7 ± 1.0	6.1 ± 0.6^bc^	6.6 ± 1.1^b^	5.5 ± 0.9^c^	7.0 ± 1.2^a^	7.0 ± 0.5^abc^	7.1 ± 1.1^ab^	7.0 ± 1.0^ab^	5.1 ± 0.6^d^	6.5 ± 1.5^b^
5	6.8 ± 1.4^bcde^	7.1 ± 0.9	6.2 ± 1.4	5.9 ± 1.3	6.5 ± 1.3^b^	5.0 ± 1.3^d^	4.1 ± 0.5^f^	4.5 ± 1.3^d^	6.7 ± 1.0^bcd^	6.8 ± 1.1^bc^	6.8 ± 1.1^bc^	5.5 ± 1.1^c^	5.0 ± 1.5^d^
6	7.0 ± 1.2^abcde^	7.1 ± 0.9	6.2 ± 1.5	6.1 ± 1.5	5.5 ± 1.1^d^	6.5 ± 1.1^b^	5.1 ± 0.8^d^	7.0 ± 0.6^a^	6.8 ± 1.2^bcd^	6.7 ± 1.2^bc^	6.7 ± 1.1^bc^	5.5 ± 1.2^c^	7.2 ± 0.8^a^
7	7.0 ± 1.3^abcd^	7.2 ± 0.7	6.2 ± 1.4	6.1 ± 1.4	6.1 ± 1.2^bc^	6.5 ± 1.4^b^	6.5 ± 1.2^a^	6.5 ± 0.7^b^	6.0 ± 1.3^e^	5.9 ± 1.0^d^	6.0 ± 1.1^d^	7.0 ± 1.0^ab^	6.5 ± 1.1^b^
8	6.5 ± 1.1^e^	7.2 ± 0.8	6.5 ± 1.6	6.1 ± 1.6	6.1 ± 1.3^bc^	6.5 ± 1.4^b^	4.5 ± 1.0^e^	6.5 ± 1.0^b^	7.3 ± 1.3^a^	7.3 ± 0.8^a^	7.3 ± 1.0^a^	4.6 ± 0.9^e^	6.1 ± 1.2^c^
9	7.2 ± 0.9^ab^	7.3 ± 0.8	6.4 ± 1.4	6.0 ± 1.5	7.0 ± 0.4^a^	6.3 ± 1.4^b^	5.6 ± 1.1^c^	6.7 ± 1.0^ab^	7.1 ± 0.9^ab^	7.0 ± 1.2^ab^	7.0 ± 1.1^ab^	6.7 ± 1.2^b^	6.0 ± 1.1^c^
10	7.3 ± 1.0^a^	7.3 ± 0.8	6.5 ± 1.4	6.1 ± 1.2	7.0 ± 0.4^a^	6.5 ± 1.2^b^	5.6 ± 1.3^c^	6.8 ± 1.4^ab^	6.9 ± 0.9^abcd^	7.0 ± 1.2^ab^	6.8 ± 0.9^bc^	6.8 ± 1.2^b^	6.0 ± 1.0^c^
p-value	0.003	0.225	0.543	0.708	< 0.001	< 0.001	< 0.001	< 0.001	< 0.001	< 0.001	< 0.001	< 0.001	< 0.001

The different superscript letter in the same column indicated the significant difference (p < 0.05).

NS superscript indicated the non-significant difference (p > 0.05) within the same column.

1 = dislike extremely, 2 = dislike very much, 3 = dislike moderately, 4 = dislike slightly, 5 = neither like nor dislike, 6 = like slightly, 7 = like moderately, 8 = like very much, 9 = like extremely.

The significant effect on the MSIC appearance can be attributed to the addition of BPP and CP which can affect the physical properties of ice cream mixed with spices. The differences in appearance can happen because the uneven dispersion of spice powder into the ice cream matrix affects the appearance and causes it to be significant to the panels' perception, as suggested in the research of Tridevi et al.^28^ on the manufacture of ice cream using basil powder as a flavoring agent. Additionally, the uneven dispersion of spice powder into MSIC can also affect flavor perception. There was a wide range of sensory rating scores of cinnamons and pepper flavor from 5.0–7.2 and 4.1–6.3. These results suggest that the increase of spiced powder into MSCI can cause the sensory rating score to increase, however, an exceedingly high increase of mixed spice can decrease the liking score of the product because of an excessively strong flavor^20,28^.

Other sensory responses that were affected by BPP and CP were melting in mouth, creaminess, oiliness, and viscosity, which provided rating scores in the range of 6.0–7.3, 6.0–7.3, 5.9–7.1, and 5.0–7.0, respectively. Increasing BPP and CP altogether affected all mentioned attributes above to be increased similarly to melting rate and overrun of the MSIC. The quantity of spice powder added into the ice cream mixture can increase firmness and viscosity because the interaction of spice powder and water in the mixture causes large ice crystal formation which affects the texture state and retards the melting of ice cream which indicated the correlation of physical properties and sensory quality of MSIC^23^.

Since BPP and CP contain compounds that give distinctive sweet and spicy flavors^35,36^. Consequently, the variation of BPP and CP mixed into MSIC affected the sweetness and spiciness significantly. The ratio of BPP and CP was vital to the cinnamon and pepper flavor in MSIC. The mixed ratio of BPP and CP at 1:1 resulted in the highest sensory rating score of cinnamon and pepper flavor. When the ratio of BPP and CP were deviating from 60 to 96% the preference score significantly decreased. This suggests that a suitable proportion of spice powder applied in a food product can help balance the flavor compounds appropriately, increasing the preference rating score of sweetness, spiciness, and overall liking in MSIC.

### Model fitting for the optimization of BPP and CP in MSIC

**Figure 1:**
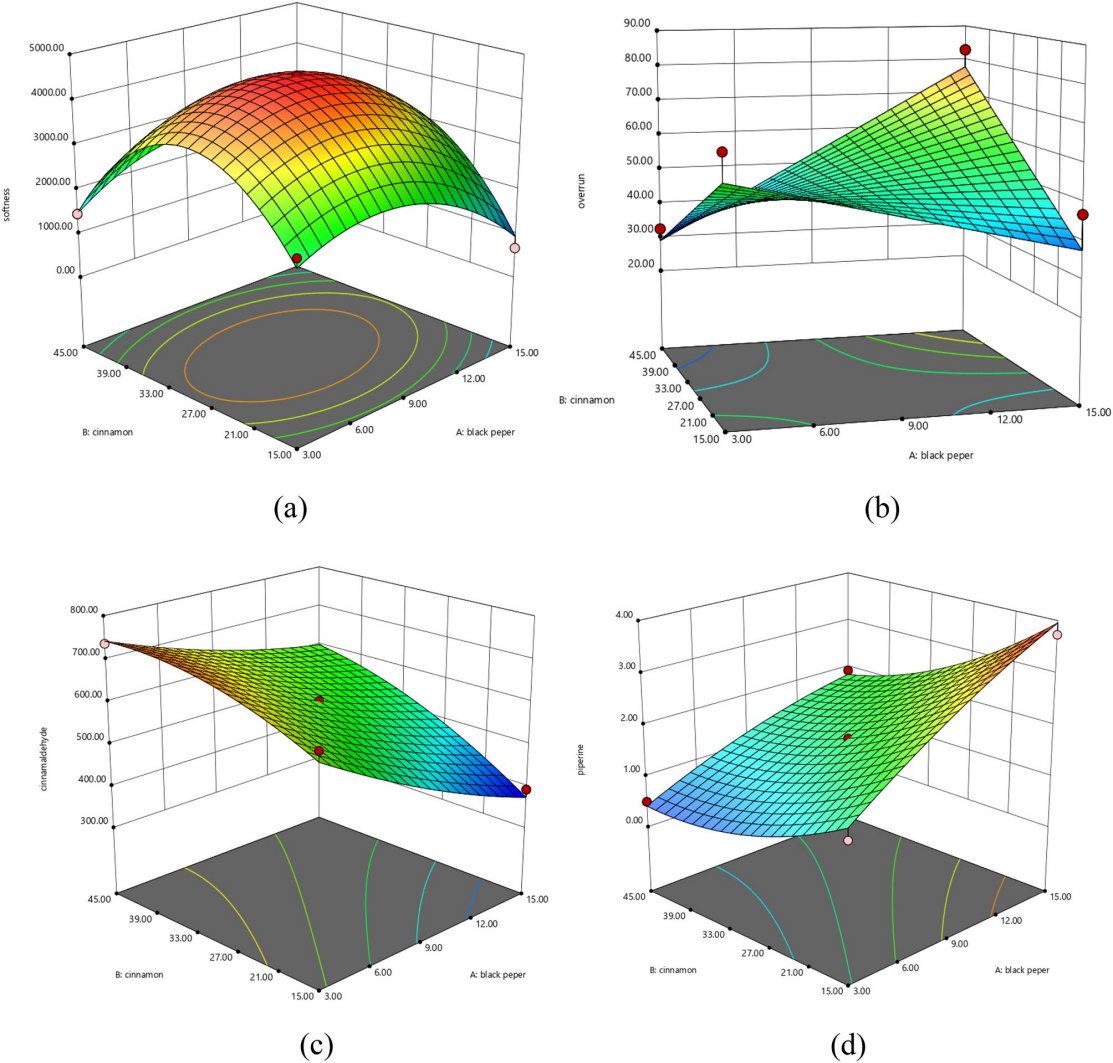
The response surfaces demonstrate regression model of MSIC using BPP (A) and CP (B); (**a**) softness, (**b**) overrun, (**c**) cinnamaldehyde, and (**d**) piperine.

**Figure 2:**
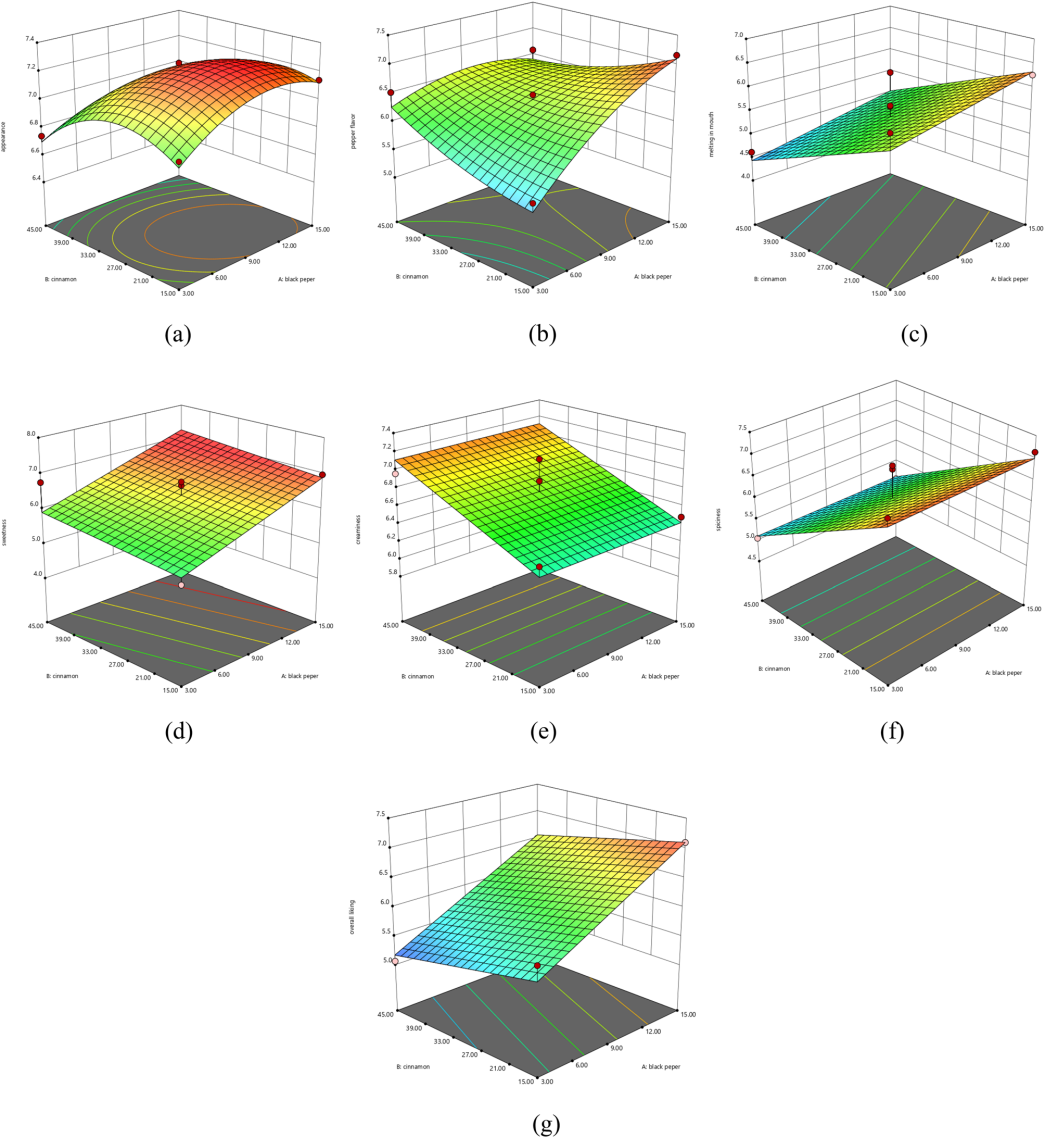
The response surfaces demonstrate regression model of MSIC using BPP (A) and CP (B); (**a**) appearance, (**b**) pepper flavor, (**c**) melt in mouth, (**d**) sweetness, (**e**) creaminess, (**f**) spiciness, and (**g**) overall liking.

The MSIC properties were carried forward to optimize the amount of BPP and CP using RSM (Figs. 1 and 2). The firmness, overrun, cinnamaldehyde, piperine, and sensory responses (appearance and pepper flavor) were fitted to a quadratic model with interaction whereas other sensory responses (melt in mouth, sweetness, creaminess, spiciness, and overall liking) were fitted to a linear model regression as shown in Table 4, where A and B denote BPP and CP.

**Table 4:** Regression equation of significant responses from MSIC using BPP and CP.

Responses	Regression equation	R-square	Significant level
Softness	− 4273.61 + 349.87*A + 493.87*B + 3.72*AB − 28.02*A^2^ − 8.78*B^2^	0.9226	0.0049
Overrun	87.02 + 4.64*A + 1.56*B + 0.19*AB	0.8403	0.0084
Cinnamaldehyde	571.58 − 35.45*A + 12.21*B + 0.32*AB + 0.51*A^2^ − 0.16*B^2^	0.9692	0.0008
Piperine	2.20 + 0.33*A − 0.12*B − 0.003*AB − 0.005*A^2^ + 0.002*B^2^	0.9644	0.0011
Appearance	5.99 + 0.11*A + 0.06*B − 0.0001*AB − 0.004*A^2^ − 0.001*B^2^	0.9559	0.0016
Pepper flavor	4.49 + 0.33*A + 0.003*B − 0.004*AB − 0.01*A^2^ + 0.0001*B^2^	0.8143	0.0274
Melting in mouth	6.23 + 0.05*A − 0.04*B	0.6972	0.0153
Sweetness	5.06 + 0.11*A + 0.01*B	0.6331	0.0299
Creaminess	6.06 + 0.0003*A + 0.02	0.7633	0.0065
Spiciness	7.95 − 0.0003*A − 0.06*B	0.7981	0.0377
Overall liking	5.63 + 0.12*A − 0.02*B	0.9448	< 0.0001

*A* black pepper powder, *B* cinnamon powder.

The regression equation of firmness, overrun, cinnamaldehyde, and piperine from Table 4 were employed to generate the response surface as shown in Fig. 1. The BPP and CP affected firmness and overrun in opposite directions. Increasing the BPP and CP increased the firmness and decreased the overrun. The regression equation of piperine and cinnamaldehyde shows that increasing BPP and CP also causes an increase of piperine and cinnamaldehyde as the major volatile compounds of BPP and CP are piperine and cinnamaldehyde^7,8,37^. The R-square value of the significant physicochemical responses was in the range of 0.8403–0.9692, with the significant level being in the range of 0.0008–0.0084.

The regression equation of sensory responses from Table 4 was also employed to create the response surface as shown in Fig. 2. Increasing both BPP and CP can trigger the consumer preference in pepper flavor, sweetness, and creaminess to be increased whereas the decreasing of either BPP or CP separately can only affect the consumer preference in spiciness and melt in mouth to be increased individually.

The reason for this incident is that only a small amount of spice can affect the intensity of aroma and flavor. An exceedingly high amount of BPP will affect the preference for spiciness whereas an exceedingly high amount of CP can affect the melt in mouth preference due to the presence of irritation particles in the mouth and throat.

The optimum formulation for the MSIC consists of BPP and CP based on the significant response as shown in Table 4. The criteria for the optimized amount of BPP and CP were determined from the highest value of firmness, overrun, cinnamaldehyde, piperine content, and sensory rating score. The optimized BPP and CP in MSIC were 15.00 g and 34.00 g, which provided firmness, overrun, cinnamaldehyde, piperine, appearance, melt in mouth, pepper flavor, sweetness, creaminess, spiciness, and overall liking at (3210.65 ± 105.74 g.force), (61.63 ± 0.60%), (555.41 ± 10.37 µg/g sample), (2.28 ± 0.02 µg/g sample), (7.1 ± 1.1), (6.0 ± 0.9), (6.7 ± 8), (6.9 ± 0.8), (7.0 ± 0.8), (6.0 ± 1.1), and (6.9 ± 0.8). The approximation error between the prediction and observed value of the significant physicochemical properties and sensory responses of the MSIC was in the range of 0.05–9.09%. An error percentage lower than 10% indicated that the acquired BPP and CP for MSIC showed confidence abutting to the predicted value.

### Consumer acceptance of optimized MSIC

The MSIC with optimized BPP and CP was subjected to evaluation for consumer acceptance. The preference rating score from consumer acceptance (n = 400) showed that the MSIC exhibited an acceptance rating score in the range of ‘like slightly’ to ‘like moderately’ (6.0–7.2) (Supplementary Fig. A) which indicated that the ice cream with BPP and CP had desirable sensory attributes with the novelty of black pepper and cinnamon. In the present study, the optimized BPP and CP provided uniform color with pleasant flavor which is an essential determinant for consumer acceptance and preference. The optimized BPP and CP also affected the MSIC to carry over desirable textural attributes as initial firmness with slight resistance and a distinct smoothness and creaminess in the mouth, as it melts. These were consistent with studies on the development of ice cream fortified with herb, spices, and its extract that provided the preference rating score in the range of like moderately to highly like since herbs, spices, and its extract can be sufficient to improve the palatability and texture of the base product, which was frozen^5,20,38,39^. Besides, the addition of herbs, spices, and their extract also increased taste intensity which affected the favorable of consumers toward the optimized MISC.

## Conclusion

This research concludes that the application of RSM on the optimization of BPP and CP in MSIC can create appealing properties and product acceptability. The BPP and CP can also provide exceptional flavor and bioactive compounds which can be used as favorable ingredients for dairy products to obtain compatibility products with herbs and spices. The outcomes from this research can be applied to develop dairy products innovatively fortified with herbs and spices to increase potential consumption and deliver health-beneficial compounds to consumers.

## Supplementary Information

Supplementary Figure 1.

Supplementary Table 1.

## Data Availability

The datasets generated during and/or analyzed during the current study are available from the corresponding author on reasonable request.
