# Supplementary material for: Determination for a suitable ratio of dried black pepper and cinnamon powder in the development of mixed-spice ice cream
Source: Sci Rep. 2022 Sep 6;12:15121. doi: 10.1038/s41598-022-19451-7 (PMC9448764; doi:10.1038/s41598-022-19451-7)
Supplement: Supplementary file 1 — Supplementary Figure 1. [file 41598_2022_19451_MOESM1_ESM.docx]

**Supplementary Fig. A.** The consumer acceptance (n=400) of MSIC with optimised BPP and CP.
