# Supplementary material for: Determination for a suitable ratio of dried black pepper and cinnamon powder in the development of mixed-spice ice cream
Source: Sci Rep. 2022 Sep 6;12:15121. doi: 10.1038/s41598-022-19451-7 (PMC9448764; doi:10.1038/s41598-022-19451-7)
Supplement: Supplementary file 2 — Supplementary Table 1. [file 41598_2022_19451_MOESM2_ESM.docx]

**Table A**

Formulation of Black pepper powder (BPP) and Cinnamon powder (CP using Central Composite Design with 2 centerpoints

| **Treatment** | **Coded** | | **Actual** | |
| --- | --- | --- | --- | --- |
|  | **BPP** | **CP** | **BPP (gram)** | **CP (gram)** |
| **1** | -1 | -1 | 3.00 | 15.00 |
| **2** | 1 | -1 | 15.00 | 15.00 |
| **3** | -1 | 1 | 3.00 | 45.00 |
| **4** | 1 | 1 | 15.00 | 45.00 |
| **5** | -α | 0 | 0.51 | 30.00 |
| **6** | α | 0 | 17.49 | 30.00 |
| **7** | 0 | -α | 9.00 | 8.79 |
| **8** | 0 | α | 9.00 | 51.21 |
| **9** | 0 | 0 | 9.00 | 30.00 |
| **10** | 0 | 0 | 9.00 | 30.00 |
